# High-Performance Handball Player’s Time-Motion Analysis by Playing Positions

**DOI:** 10.3390/ijerph17186768

**Published:** 2020-09-17

**Authors:** Carmen Manchado, Juan Tortosa Martínez, Basilio Pueo, Juan Manuel Cortell Tormo, Helena Vila, Carmen Ferragut, Francisco Sánchez Sánchez, Sonia Busquier, Sergio Amat, Luis Javier Chirosa Ríos

**Affiliations:** 1 Faculty of Education, University of Alicante, 03690 San Vicente del Raspeig, Spain; carmen.manchado@ua.es (C.M.); juan.tortosa@gcloud.ua.es (J.T.M.); basilio@gcloud.ua.es (B.P.); jm.cortell@gcloud.ua.es (J.M.C.T.); 2 Faculty of Education, University of Vigo, 36905 Pontevedra, Spain; 3 Faculty of Medicine and Health Sciences, University of Alcalá, 28871 Alcalá de Henares, Spain; cferragutfiol@gmail.com; 4 Faculty of Sport Science, University of Castilla La Mancha, 45071 Toledo, Spain; Fco.Sanchez@uclm.es; 5 Department of Applied Mathematics and Statistics, University of Cartagena, 30203 Cartagena, Spain; sonia.busquier@upct.es (S.B.); sergio.amat@upct.es (S.A.); 6 Department of Physical Education and Sports, University of Granada, 18011 Granada, Spain; lchirosa@ugr.es

**Keywords:** running pace, running distance, competition load, LPS

## Abstract

The purpose of this study was to analyze the on-court demands of handball players during the European Handball Federation Champions League Final Four (VELUX EHF FINAL4) 2019 to define time–motion characteristics (played time; covered distances) both in offense and defense. Furthermore; we aimed to define position-specific demands and differences among them. Forty players from three teams were analyzed during the tournament using a local positioning system (LPS) for the first time in top handball. Players covered similar distances both in offense (1388.28 ± 2627.08 m), and in defense (1305.47 ± 5059.64 m) and remained on court for a similar average time (15.69 ± 8.02 min and 15.40 ± 8.94 min respectively). When locomotion activities were normalized according to the time they spent on court; significant differences were found for defense compared to offense in walking (+20%; *p* < 0.000; Cohen’s effect size (ES) = 1.01) and jogging (−29.6%; *p* = 0.000; ES = 0.90), as well as a tendency for high-intensity running (+ 25.2%; *p* = 0.077; ES = 0.31). Per playing position; center and left back (CB = 94.86 ± 10.98 m·min^−1^; LB = 96.55 ± 24.65 m·min^−1^) showed the highest running pace in offense and mid-left; front center defender and outside right for the defense (ML = 90.38 ± 30.16 m·min^−1^; FCD = 87.04 ± 14.94 m·min^−1^; OR = 89.64 ± 34.93 m·min^−1^). In conclusion; profile differences existed among players’ position activity; both in offense and defense; which should be taken into account when designing specific physical training programs

## 1. Introduction

Handball is an Olympic sport, belonging to so-called team sports. It is characterized by fast transitions between offensive and defensive actions during the game with the ultimate objective of scoring a goal [1,2]. To this end, offensive players (six field players and one goalkeeper) attempt to create spaces that allow them to throw the ball towards the goal in advantageous conditions, while the defense tries to avoid it, causing a great amount of physical confrontations between players [2]. These attack phases in handball are dynamic, characterized by fast movements and a high frequency of fast passes, so physical demands are important [1]. Furthermore, these physical demands are not only the same if the team is in the offensive or defensive phase and even if the player plays in one position or another [3,4,5].

The very nature of the game implies that players must be physically trained to maintain the game’s speed and intensity throughout a match [4,6,7,8], whether they play in offense or defense. Therefore, knowing and understanding the sport’s physical demands (distances, speeds, intensities) [1], as well as technical–tactical actions [4,7] (passes, throws, jumps, marking, change of direction, etc.) is essential to correctly plan players’ training. [1,8]. All these elements are of great importance in handball and are also closely related to each other, which makes handball a particularly complex sport [4,5,9].

Likewise, it is important to note that the playing position, the game phase (offense or defense), as well as the team’s playing style can lead to big differences in each player’s physical demands. Therefore, the physical load cannot only be determined generally, but according to each player’s specific position on the court both in offense and defense [4,6,10]. All this information could help coaches to better individualize training loads and thereby improve performance [4,6].

This necessity to understand handball’s physical characteristics has raised great interest among researchers who have studied these demands using different methodologies [1]. The most widely used method has been time–motion analysis, based on observing players in the competition followed by an analysis of a video, taken with one camera [11,12] or two cameras [13]. The video-recorded matches are analyzed and the actions encoded. However, this method is time-consuming and depends on a subjective analysis of the observer, thus not being an objective or precise method when determining the different locomotion speeds.

Notwithstanding, no method exists to date that allow one to accurately measure the physical and physiological demands of handball players during the competition. In order to overcome this gap, the European Handball Federation (EHF), Select^®^ and Kinexon^®^ jointly developed the Kinexon^®^ tracking system for handball players (Kinexon: München, Germany; Select Sport 1947: Glostrup, Denmark) in addition to a monitored ball, the iball, which has been recently validated [14] and used in studies on handball [15] and other team sports [16]. This technology provides us with values regarding movements, accelerations, changes of direction, jumps, as well as data on the speed at which the ball is transferred (game speed) and the speed and position of the throws in real time, opening up new possibilities in the study of handball competition requirements [16]. With this fully automatic tracking system, the inconveniences mentioned for the conventional time–motion analysis are solved.

Despite a great interest in understanding the requirements of high-level players, only a few studies have focused on analyzing the real demands of an elite handball competition in male handball [3,13,17,18]. Cardinale, Whiteley, Hosny, and Popovic [3], studied players’ movements during the men’s world championship using three cameras, and provided new data on players’ movements (distances and intensities) during the match. They concluded that there was no significant difference in terms of distance covered in different locomotion categories, but they did not distinguish between offense—and defense—specific playing positions. In the same line, González de Haro [17] reported the analysis of only one match with Global Positioning System devices (WIMU PRO™, Realtrack Systems S.L.: Almeria, Spain). These researchers [3,17] concluded that specific physical conditioning is necessary to maximize performance of handball players and minimize the occurrence of fatigue.

To the best of our knowledge, no study has been conducted considering in detail the two phases of the game, offense and defense, and analyzing all the playing positions by using a technology that allows load individualization and automation, a local positioning system (LPS). Better knowledge of on-court demands of handball players at the highest level is necessary to improve the individualization of physical preparation [3,6,7,17,18].

Thus, the aim of this study was to analyze on-court demands of handball players during the VELUX EHF FINAL4 to define time–motion characteristics (played time, covered distances) both in offense and defense, including position-specific demands and differences among them.

## 2. Materials and Methods

### 2.1. Subjects

Data were obtained from players participating in the VELUX European Handball Federation (EHF) Champions League Final Four 2019/20, held in Cologne (Germany). The teams that participated in the Final Four were FC Barcelona (Spain), Telekom Veszprém (Hungary), HC Vardar (The Republic of North Macedonia), and KS Kielce (Poland). Barcelona’s players were not included in the study because their sensors were not placed properly, causing interferences in the signal and thus unreliable data. Dainis Krištopāns (HC Vardar) did not wear the sensors during the games so his data were not available for the analysis either. Finally, 40 players were analyzed during both semifinals, the final championship game and the bronze medal game. Goalkeepers were excluded from the analysis as distance and motion characteristics do not reflect their performance needs. Anthropometric characteristics and the age of the players are presented in Table 1. This information was collected from the official statistical data provided by the EHF.

### 2.2. Instruments

**Figure 1:**
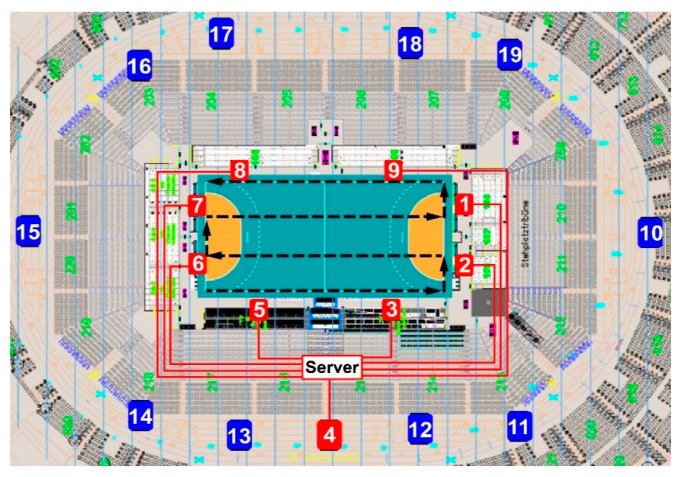
Local positioning system (LPS) setting: nine antennae connected to the server in red locations; ten reference antennae (anchors) in blue locations; meander path inside the field followed to check calibration accuracy (black discontinued line).

The players’ position data were collected through a Local Positioning System (LPS) (Kinexon Precision Technologies, Munich, Germany), which has been recently validated [7] and used in studies on team sports [8,9], showing adequate between-device reliability (coefficient of variation around 5%) when compared to well-known systems such as GPS. Firmware versions and application software versions corresponded to the latest releases on the testing date (August 2019). Figure 1 shows the setting of the 9 antennae around the playing field, connected via ethernet to the main server, and 10 anchor antennae distributed at 3 different levels above the ground in the Lanxess Arena.

The LPS system was installed, calibrated, and checked for accuracy by a technician who worked for the manufacturer as follows: The exact position of the anchors in reference to the playing field was measured (blue numbered positions in Figure 1). Then, the anchor positions and the playing field position and size were transmitted to the Kinexon application. The location of one sensor at pre-defined positions (corner, penalty line, center point) was checked. In addition, two paths were followed to test the data quality and calculated distance—walking on the sideline and walking on a meander inside the field (black discontinued line in Figure 1). The devices worn by players comprised a sensor (player tag) positioned between the player’s shoulder blades using a pouch sewn onto the player’s jersey. The functionality of the sensors was tested in the venue by randomly walking and checking if signals were received from all units with adequate signal strength. These sensors transmit time signals via radio-technology to the antennae, which send signals via a wide local area network (WLAN) to local static base stations at known locations. A player’s momentary position is determined via 20 Hz frequency by calculating the time-of-flight (TOF) of ultra-wide-band radio signals traveling from the transmitter to the base stations, which calculate the actual 2D position of the devices within the playing field. Subsequently, instantaneous speed, i.e., scalar magnitude of velocity, as per the rate of change in horizontal x, y positions, and acceleration, as per the rate of change in speed, are derived by calculating the difference between two consecutive positions, i.e., approximating the derivative of the player’s position. The raw position and speed data are then filtered and smoothed by means of a Kalman filter for position data and an exponential moving average with a window length of 1 s for speed and position data. Data were split into offensive and defensive moments of play automatically. To this end, there was automatically a change from offensive to defensive for the team and vice versa at the moment where the ball possession changed. The respective offensive shift started with the ball possession of the team. Moreover, the system also checked if the players and the ball were moving in the direction of the opponent’s goal. In the event the ball was outside the court, the shift was interrupted. All data were analyzed using the system software (Kinexon Web Application, version 3.2.6, Munich, Germany).

### 2.3. Procedure

In this study, a descriptive observational cross-sectional study was used to examine the physical demands according to playing positions during competitive matches. This time–motion analysis is used with team [5,19] and beach handball [19], as well as with other team sport studies [20,21].

The study was approved by the EHF. The clubs signed an informed consent in the initial contract with the EHF to take part in the competition, where they accepted the rules and norms of the EHF, including their participation in different studies. The players’ data were anonymized for the purpose of this study. The players were informed of the purposes, procedures, and risks of the study and provided informed consent before the beginning of the study. All the procedures were conducted in accordance with the Declaration of Helsinki and approved by the Ethics Committee of the University of Vigo (registration number 04-719).

The variables described next were measured based on position and speed data. The distances covered during the entire match (total distance/duration of play), distances per minute during play and relative distance in established speed zones were computed. These zones were set as zone 1: standing (≤0.9 m/s), zone 2: walking (1.0–1.9 m/s), jogging (2.0–3.9 m/s), running (4.0–5.4 m/s), high-intensity running (5.5–6.9 m/s) and sprinting (≥7 m/s), in accordance with similar handball studies [3,5,18,19].

We also considered the distinction between offense (when the team was in possession of the ball) and defense (not in possession of the ball), and classified the players by their positions according to handball nomenclature in offense (left wing = LW, left back = LB, center back = CB; line player = LP; right back = RB; and right wing = RW) and defense (center back = CB; mid right = MR; mid left = ML; outside right = OR; outside left = OL; and front center defender = FCD). The descriptive analysis of the data included the mean, the range, the variance and the standard deviation.

### 2.4. Data Analysis

Graphical, analytical and numerical studies were performed using our own developed programs. The Shapiro–Wilk test was performed in order to verify the normality of the data. Group differences were determined by variance analysis (ANOVA) followed by Games–Howell or Tukey post hoc testing, or Student’s *t*-tests for independent samples, where appropriate. To determine the magnitude of each relationship, Cohen’s effect size (ES) was used with a modified classification (trivial <0.2, small 0.21–0.6, moderate 0.61–1.2, large 1.21–1.99, and very large >2.0) proposed for sports sciences [22] and used in other similar handball studies [3]. The precision of population estimates was reported as 95% confidence intervals, and statistical significance was set at *p* < 0.05.

## 3. Results

### 3.1. Time on Court, Distance Covered in Offense and Defense

**Figure 2:**
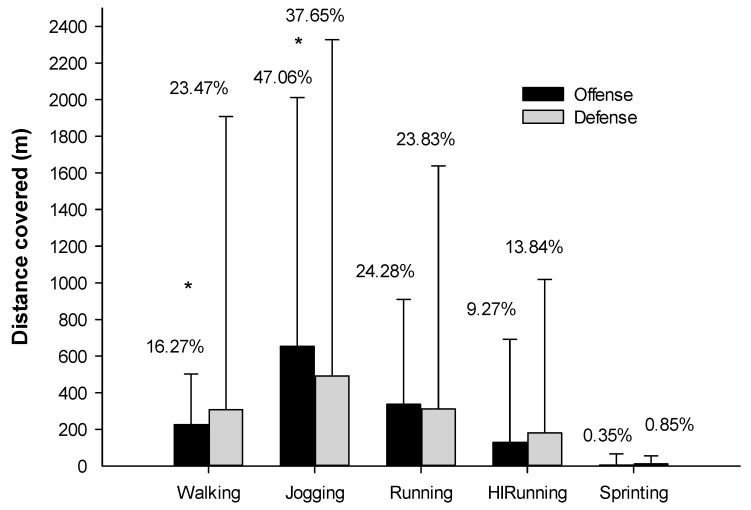
Differences in distance covered in offense and defense during different locomotion characteristics. * Statistical differences; *p* ≤ 0.05.; HIRunning: High-Intensity running

The average time on court in offense (*n* = 66) and defense (*n* = 67) during the VELUX EHF Final 4 was 15.69 min (±8.02 min) and 15.40 min (±8.94 min), respectively. The total average distance covered per player during each game in offense was 1388.28 ± 2627.08 and 1305.47 ± 5059.64 m in defense. When comparing offense and defense with regard to the absolute distances covered (Figure 2), significant differences were found in walking (*p* = 0.017; ES = 0.61) and jogging (*p* = 0.03; ES = 0.77), as well as a tendency towards high-intensity running (*p* = 0.075; ES = 0.45).

Locomotion activities were then normalized for each player according to the time they spent on court to obtain a true reflection of these demands, both for offense and defense. The running pace per game showed by the complete team in offense was 88.45 ± 20.72 m·min^−1^, walking: 13.89 ± 2.98 m·min^−1^, jogging: 40.55 ± 10.12 m·min^−1^, running: 23.65 ± 12.53 m·min^−1^, high-intensity running: 9.70 ± 9.39 m·min^−1^ and sprinting: 0.42 ± 0.94 m·min^−1^.

The running pace per game showed by the complete team in defense was 80.83 ± 27.11 m·min^−1^, walking: 17.53 ± 4.18 m·min^−1^, jogging: 28.56 ± 4.18 m·min^−1^, running: 20.49 ± 11.47 m·min^−1^, high-intensity running: 12.96 ± 11.54 m·min^−1^ and sprinting: 0.56 ± 1.29 m·min^−1^.

When comparing offense and defense, significant differences were found in walking (*p* < 0.000; ES = 1.01) and jogging (*p* = 0.000; ES = 0.90), as well as a tendency for total distance (*p* = 0.71; ES = 0.32) and high-intensity running (*p* = 0.077; ES = 0.31).

### 3.2. Positional Differences in Distance Covered and Speeds

**Figure 3:**
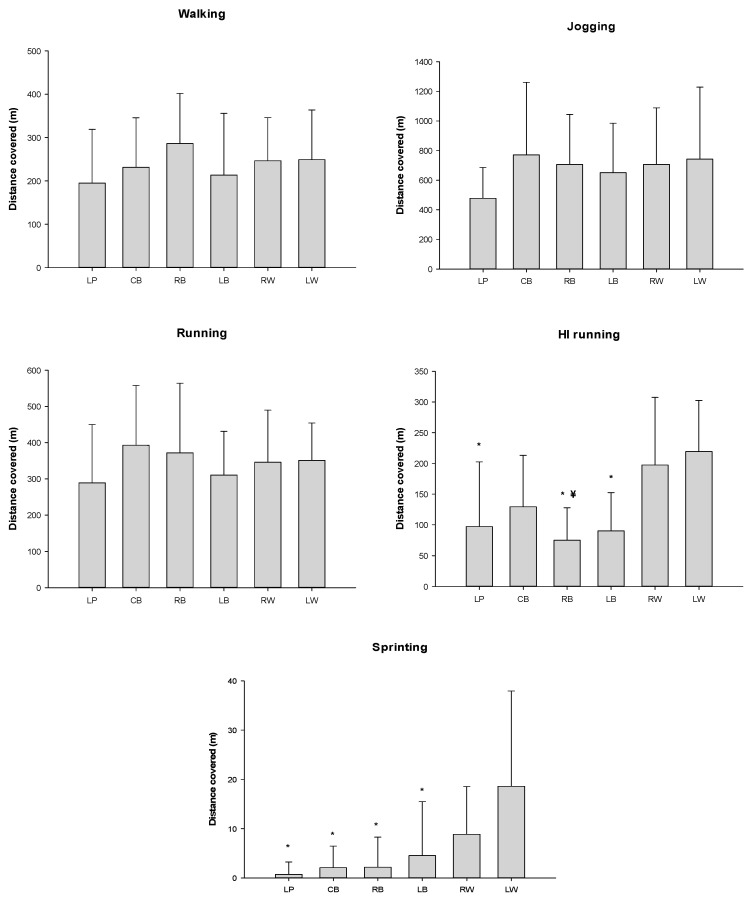
Distance covered in different locomotion category by playing position in offense. * Statistical differences with the left wing *p* ≤ 0.05; ¥ = statistical differences with the right wing *p* ≤ 0.05. Legend: left wing = LW; left back = LB; center back = CB; line player = LP; right back = RB; right wing = RW.

The distances covered in offense by playing position for each locomotion category are shown in Figure 3.

Although not many significant differences were found, high effect size values were obtained (Table 2).

**Figure 4:**
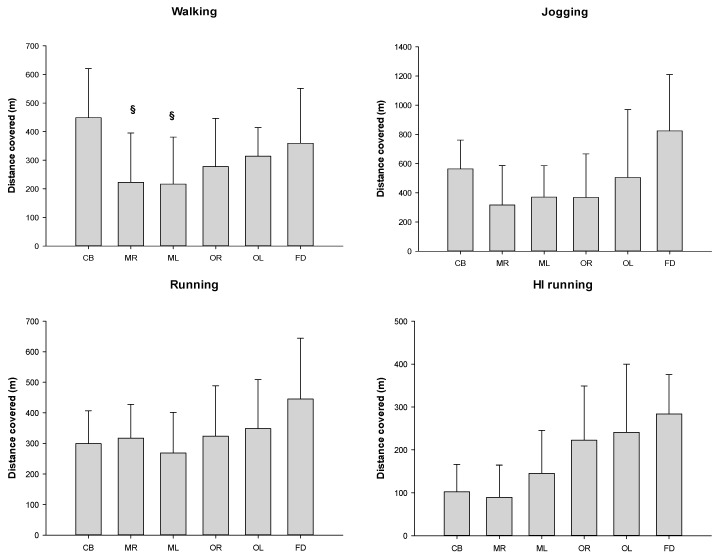

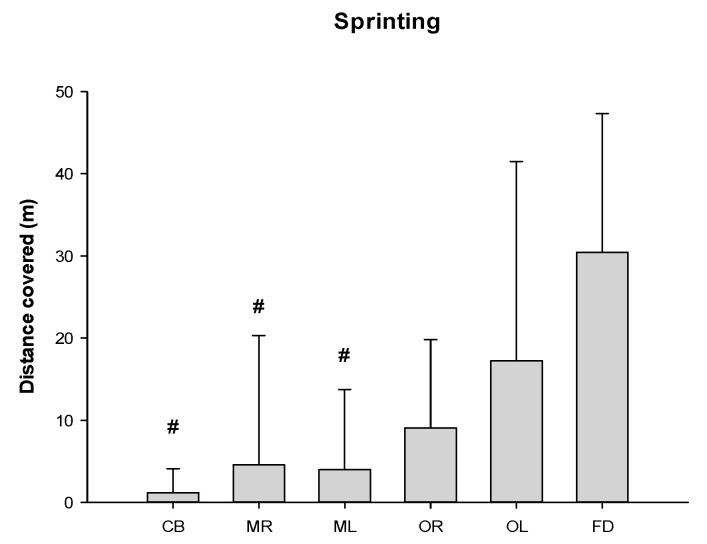
Distance covered in different locomotion categories by playing position in defense. # Statistical differences with front defender *p* ≤ 0.05; § statistical differences with center back *p* ≤ 0.05. Legend: center back = CB; mid right = MR; mid left = ML; outside right = OR; outside left = OL; front center defender = FD.

The distances covered in defense for each locomotion category by playing position are shown in Figure 4.

Furthermore, moderate, large and very large effect sizes were found in the different locomotion characteristics by playing positions in defense (Table 3).

### 3.3. Running Pace by Playing Positions

**Figure 5:**
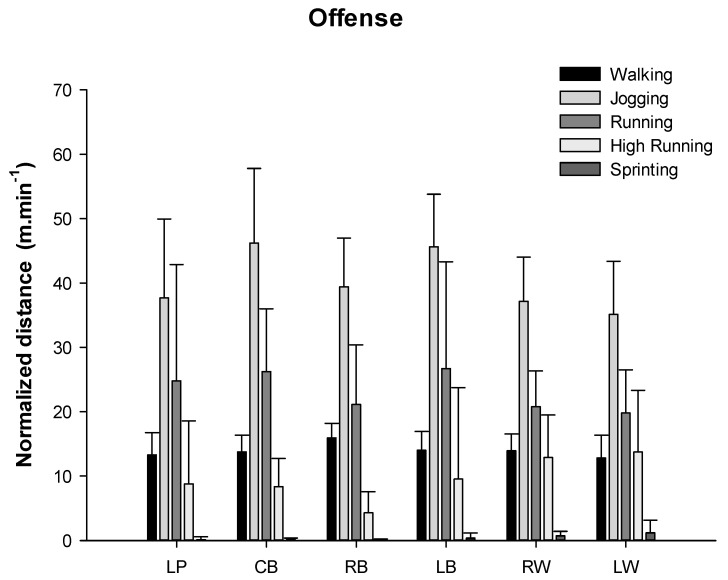
Distance covered in each locomotion category normalized according to the time spent in court in different playing positions during offense. Legend: left wing = LW, left back = LB, center back = CB; line player = LP; right back = RB; right wing = RW.

When the distance covered in each locomotion category normalized according to the time spent on court in different playing positions during offense were analyzed, the ANOVA showed significant differences for jogging (*p* = 0.029) and sprint distances (*p* = 0.045) between the different playing positions. However, the post hoc analysis did not show any statistically significant differences (Figure 5).

**Figure 6:**
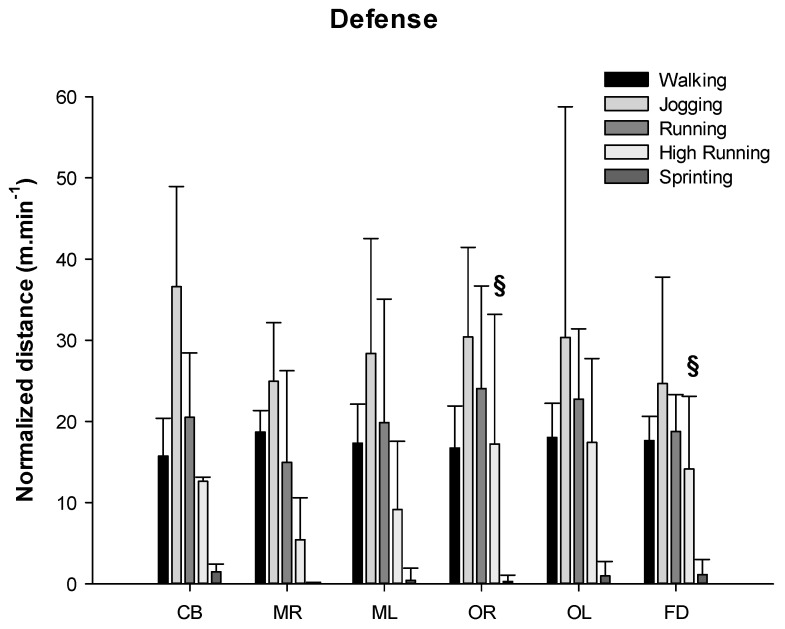
Distance covered in each locomotion category normalized according to the time spent in court in different playing positions during defense. § Statistical differences with center back *p* ≤ 0.05. Legend: center back = CB; mid right = MR; mid left = ML; outside right = OR; outside left = OL; front center defender = FD.

When the distance covered in each locomotion category normalized according to the time spent on court in different playing positions during defense was analyzed, the ANOVA showed significant differences only for high-intensity running (*p* = 0.038) between the different playing positions (Figure 6). Post hoc analysis showed significant differences in this category between the central back and the outside right (*p* = 0.016; ES = 1.19) and the front defender (*p* = 0.003; ES = 0.37), as well as a tendency between the central back and the mid left (*p* = 0.074; ES = 1.20).

## 4. Discussion

The aim of this study was to analyze on-court demands of handball players during the VELUX EHF FINAL4 to define time–motion characteristics (played time, covered distances) both in offense and defense, including position-specific demands and differences among them. Significant differences were found between offense and defense in the walking and jogging categories. In offense, significant differences were established in the high-intensity running category between LW and other playing positions. In defense, differences were also identified for CB in walking, and the FCD in sprinting when compared to other playing positions.

Several studies have analyzed handball games differentiating intensity categories [3,5,11,17,19,23,24], although they have taken into account neither all playing positions nor the different phases of the game. In this regard, there is a broad consensus among researchers on the need to establish certain categories when analyzing players’ movements, ranging from low-intensity (standing, walking, jogging), medium-intensity (running) and high-intensity (HIrunning, sprinting) situations. However, little consensus exists on the speed ranges for defining the different categories, which makes it difficult to compare between the different studies.

For the analysis of the intensity at which the player moves across the field, the categorization proposed by Cardinale et al. [3] used for the Qatar WCh 2015 study was applied. Analyzing the results by locomotion categories, we found that for the offense, the longest distance was performed in jogging (47.06%), while defenders covered a greater distance in the walking category. When comparing offense and defense, results showed a trend for significance in the HIrunning category. These results highlight the needs to differentiate the characteristics of the game phases. As we do not have more studies to compare these results, we present an analysis of the general data with respect to other research carried out.

Globally, players covered similar distances both in offense and defense. These results are in line with the study reported by Michalsik et al. [11], which also analyzed distances by phases of the game. Other studies [3,13] have analyzed these variables without differentiating the game phases. They were conducted during the final phase of top level international men´s competition, the Men’s World Handball Championships of Germany (2007) and Qatar (2015), respectively. Our results are consistent with these studies regarding the total average distances covered, being 8% higher in the Germany WCh and 1% smaller in the Qatar WCh 2015.

Other studies that analyzed men´s top national leagues showed greater covered distances than ours, ranging from 3157 m on average in the analysis of the German first division [25] to 3627 m in the main Danish league [4], or 4370 m in the main Portuguese league [12]. The problem is that, in most of these studies, standard deviations from the average are very high, thus complicating the use of this criterion as a performance control measure. A better criterion, with a practical application for coaches, is to normalize the data according to a players’ time on court [3,6]. When the data are normalized according to the time spent on court, which in the case of national leagues is larger (above 40 min) than in international competitions, the average running pace does not differ too much between the studies, that is, by about 10%. According to this criterion, at the highest level, a handball player covered between 70 and 90 m·min^−1^. This data normalization facilitates the comparison between studies and allows the coaches to dispose of a workload reference regarding locomotion activities.

Regarding time spent on court, offense and defense presented average times of 15.69 min and 15.40 min, respectively. Summing up both phases, values were similar to those presented by Luig et al. [13] and Cardinale et al. [3], being equal to 32 min and 37 min respectively, but lower that those described in national league studies [3,4,14,15], where values over 40 min were found.

Regarding the average running pace values in offense and defense (89 m·min^−1^ and 81 m·min^−1^, respectively), our results did not match those presented by Michalsik et al. [11], although the differences were less than 10%. In this line, the results showed in the different studies are very heterogeneous, possibly due to the methodology used, the instrument of control, level of play, the category and the competition analyzed.

Regarding specific positions, our main finding was that there were differences among players’ locomotion categories in each playing position, both in offense and defense, which implies a need for greater differentiation and individualization in the training load according to the different playing positions.

In the case of the offense, left wing players showed the highest covered distances in the high-intensity running and sprinting categories in relation to the other specific positions, and the right wings covered longer distances than right back players for the HIrunning category. We should bear in mind that it is possible that some statistical comparisons in our study may show no statistical significance even when the means of the two groups are quite different, because the sample is small and the SD are high. In this context, ES might be a good indicator not only of the magnitude of the changes but also of the associations that are likely to present significant differences in larger samples.

The ES in the HIrunning and sprinting categories reinforces the observed statistical differences, since they present a large and very large ES. Therefore, we can conclude that the wings have different demands for high-intensity activities than the rest of the players in the offensive phase. These results are reinforced by the idea that these players are those who are responsible for performing most of the counter-attacks or to reach position in the first wave of the fastbreak, which are the fastest actions of the offensive phase. These data are in line with previously reported data by Michalsik et al. [4] and Povoas et al. [5] for the offensive phase.

For the defensive phase, the results showed a similar behavior to that in the offense. It is the defense-specific playing positions (CBs and FCDs) that have the highest values during the defensive phase, showing high ES values. CBs have higher values in the covered distance for the walking category than those found for mid defenders. These results are consistent with the work performed by CBs during this phase, as they are the players who move depending on the area where the ball is directed, and these displacements are usually of low intensity. In the same line, when the locomotion categories were normalized according to the time spent on court, we observed that in the HIrunning category, CBs covered less distance than the ORs and FCDs, which indicates that CBs carried out most of their activities in the low-intensity categories.

In the sprinting category, the highest covered distances correspond to the FCDs, which showed significant differences with the CBs and Mid defenders, with a large and very large ES. These data are consistent with the specific role of this player, who carries out his activities mainly in the front defense line, covering the central area of the defense, moving from side to side. Again here, as an application to training, coaches should differentiate training by specific positions, for example, by creating very intense tasks for FCDs and wings.

To our knowledge, this is the first time that specific defensive playing positions have been analyzed, so these data cannot be compared. It provides a novel and in-depth knowledge of the real needs of this phase of the game.

The total distances covered by each playing position are smaller, for both phases of the game, when compared to those reported by Michalsik et al. [4]. Variations in the methodology and the different technology used, as well as the different competitions analyzed in both studies, may account for these differences. On the one hand, LPS technology that allows load individualization and automation through micro sensors [16] was used for this study. However, Michalsik et al. [4] performed a manual estimation of intensities based on distance references on the court, following the player’s individual monitoring with a camera. Differences also existed between these two studies regarding the time spent on the court. Danish players stayed clearly longer on court in all cases. This may be because we are comparing a national league with a European final league tournament. The game-sharing times can be altered by the number and quality of players taking part in the Velux EHF Final 4, which gathers the best teams and players in the world, as well as the nature of the competition (final phase of the biggest club tournament). Therefore, the time sharing, a larger use of rolling substitutions as well as more rotations to maintain the intensity of the game may be greater than in a national league.

Other studies have also analyzed the playing positions in men’s championships with senior high level players, but have neither differentiated the phases of the game nor analyzed each individual playing position. Some of these studies have also shown greater high intensity values by the wing players compared to the rest of the playing positions [13,25].What seems to be clear is that, in general, studies that analyze the maximum distance covered per playing position varies widely, making a comparison with our study difficult because of the different procedures used [3,12,13,25].

A trend that can be observed in the available studies is that regardless of the category, procedure used, level of play or gender, all studies analyzing locomotion activities in handball have in common the differentiation of loads according to positions [3,5,6,10,19,25]. For this reason, given the great variability according to playing position, we propose to differentiate the physical work according to the role adopted in the game, in line with the conclusions of most studies.

Several limitations were found that made it difficult to discuss this study and compare it to others. The first is the small number of works focusing on the playing load in high-level men’s professional handball in final tournaments, such as the Velux EHF Final 4. Moreover, it is complicated to compare studies because of the lack of unified criteria to determine locomotion categories. It would be necessary and essential to standardize criteria so that they could be taken into consideration in future studies. Other limitations present in the work are related to the lack of development of multivariate analysis techniques. Additionally, only one championship has been analyzed, corresponding to a high performance level in the senior men’s category.

Further studies will be needed to deepen our knowledge of handball’s total load through the individualized use of sensor technology (EPTS), which allows us to learn about the other previously mentioned parameters. In addition, it would be advisable to combine the advances in the physical understanding of the game with its impact on the game’s technical–tactical component. Furthermore, there is also a need for extending the analysis to other competitions, categories and gender.

## 5. Conclusions

Offensive players covered longer distances in the jogging category and defensive players in the walking category. Profile differences existed among players’ position activity, both in offense and defense. In fact, more activity in high-intensity categories was found for wing players in offense. In the case of defense, it was the CB that covered the largest distances in low-intensity categories, and the FCD covered most of the distance in high-speed categories.

### Practical Applications

Our findings suggest the need to differentiate the training load specifically for each position, and differentiate between the phases of the game, creating specific exercises, that is, very short work (less than 2 m of displacement) involving high-intensity movements (above 5 m^−1^) and repeated in a random way over time, with high active rest time between sets. For example, you can do integrated training with simulated game situations, where FCDs in offense and LWs in defense have greater involvement. Another possibility is to design tasks that raise the fatigue threshold at each position and phase to check their impact on the game. These integrated exercises can also include explosive resistance training that improves performance in decisive final actions such as 1v1, blocks, etc.

In addition, knowing the specific load of a top-level tournament will allow coaches to determine the maximum levels of physical requirements in elite handball and set them as references based on the category. Furthermore, knowing that the different demands for the playing positions are differentiated will allow coaches to individualize and plan their workouts accordingly, as well as consider it in the match load dosing and in players’ substitutions, for example, if possible, giving more rest to the LWs and the FCDs to maintain the level of intensity.

At a high performance level, coaches should work to improve training control. The normalization of locomotion activity data allows disposing of a workload reference, in addition to facilitating the comparison between sessions. Very little information is available about the demands of the game in the different national leagues. Currently, the system is only being used in the German Bundesliga. The use of the system in the VELUX EHF Champions league would undoubtedly provide us with new relevant information about the highest competition among European clubs.

In the future, studies could be carried out to analyze players’ rotations in offense and defense. In addition, future research should relate workload on the court to the workload outside the court, such as in the fitness room. Finally, it is also possible that the results obtained in this study are useful for the future design of more specific physical tests related to the demands of the game.

## Figures and Tables

**Table 1:** Physical characteristics of the players (Mean ± Standard Deviation).

Teams	*n*	Height (cm)	Body Mass (kg)	BMI (kg/m^2^)	Age (Years)
TELEKOM VESZPRÉM	14	193.0 ± 8.8	92.9 ± 13.6	24.8 ± 1.8	31.0 ± 4.2
HC VARDAR	13	190.2 ± 10.4	90.5 ± 14.3	24.9 ± 2.4	29.7 ± 4.2
KS KIELCE	13	190.1 ± 6.4	90.1 ± 9.9	24.9 ± 2.1	28.2 ± 6.1
Total	40	191.1 ± 8.6	91.2 ± 12.5	24.8 ± 2.1	29.7 ± 4.9

Legend: BMI = Body mass Index.

**Table 2:** Effect sizes in different locomotion categories by playing position in offense.

Playing Positions	Walking	Jogging	Running	High Running	Sprinting
LP		CB 0.80			
		RB 0.86			
		LB 0.61			
		RW 0.76			
		LW 0.76			
CB			LP 0.63	LB 0.54	
			LW 0.57	RB 0.75	
RB	LP 0.75				
	LB 0.54				
LB					
RW				LP 0.93	LP 1.19
				LB 1.23	CB 0.90
				RB 1.34	RB 0.79
				CB 0.69	
LW				LP 1.25	LP 1.44
				CB 1.07	CB 1.24
				LB 1.81	RB 1.11
				RB 2.04	LB 0.95

Legend: left wing = LW; left back = LB; center back = CB; line player = LP; right back = RB; right wing = RW.

**Table 3:** Effect sizes in different locomotion categories by playing position in defense.

Playing Positions	Walking	Jogging	Running	High Running	Sprinting
CB	MR 1.31	OR 0.77		ML 0.68	
	ML 1.38	MR 1.05		OR 1.19	
	OR 1	ML 0.93		OL 1.26	
	OL 0.90	FD 1.11		FD 2.65	
	FD 0.51				
MR	OL 0.61	OL 0.52.	OR 0.65		
	FD 0.77		OL 0.84		
ML	OL 0.65				
	FD 0.84				
OR					
OL					CB 1.07
					ML 0.85
					MR 0.64
FD		MR 1.74	MR 1.46	OR 0.50	CB 4.21
		ML 1.89	CB 1.17		MR 1.62
		OR 1.45	ML 1.04		ML 2.46
		OL 0.71	OR 0.71		OR 1.80
			OL 0.56		OL 0.57

Legend: center back = CB; mid right = MR; mid left = ML; outside right = OR; outside left = OL; front center defender = FD.
